# Quadriceps and hamstring tendon autografts in ACL reconstruction yield comparably good results in a prospective, randomized controlled trial

**DOI:** 10.1007/s00402-021-03862-8

**Published:** 2021-03-19

**Authors:** Hauke Horstmann, Maximilian Petri, Uwe Tegtbur, Gernot Felmet, Christian Krettek, Michael Jagodzinski

**Affiliations:** 1 Department of Trauma, Hannover Medical School (MHH), Carl-Neuberg-Str. 1, 30625 Hannover, Germany; 2 Department of Orthopaedic Surgery, Hannover Medical School, Anna-von-Borries-Str. 1-7, 30625 Hannover, Germany; 3 Department of Sports Medicine, Hannover Medical School (MHH), Carl-Neuberg-Str. 1, 30625 Hannover, Germany; 4 ARTICO Sportklinik, Hirschbergstrasse 25, 78045 Villingen-Schwenningen, Germany

**Keywords:** ACL reconstruction, Quadriceps tendon, Hamstring tendon, Knee

## Abstract

**Introduction:**

Comparable data of functional outcomes of anterior cruciate ligament reconstruction using either hamstring- or quadriceps tendon grafts is controversial. This prospective, randomized controlled trial aims to provide data comparing both grafts regarding the functional outcome.

**Materials and methods:**

A two centre trial involving symptomatic patients 18 years of age or older with an anterior cruciate ligament tear was conducted. We randomly assigned 27 patients to quadruple hamstring tendon reconstruction and 24 to quadriceps tendon reconstruction. The patients were evaluated preoperatively, at 3, 6, 12 and 24 months post-surgery. The primary outcome parameter was the side-to-side knee laxity measured with an arthrometer. Secondary outcomes included results in the International Knee Documentation Committee (IKDC) and Lysholm Scores and isokinetic testing of strength in knee extension and flexion.

**Results:**

Forty-four patients (86%) completed the 2-year follow-up. There was significantly improved knee stability at all time intervals with no difference between the two study groups. The manual side-to-side displacement improved by 4.7 ± 3.0 mm in patients with hamstring tendon reconstruction and 5.5 ± 2.9 mm in patients with quadriceps tendon reconstruction. In addition, muscle strength and outcome scores (IKDC and Lysholm Score) did not show any differences between the hamstring tendon group and the quadriceps tendon group. Patients in the hamstring tendon group returned to their pre-injury activity level after 95.2 ± 45.5 days while patients in the quadriceps tendon group needed 82.1 ± 45.6 days.

**Conclusion:**

Quadriceps and hamstring tendon autografts yield comparably good results in primary anterior cruciate ligament reconstruction.

## Introduction

Injury of the anterior cruciate ligaments (ACL) represent the highest burden for sports related disability [24, 33]. ACL reconstruction is recommended for both chronic instabilities and athletes that wish to return early to pivoting sports and fail conservative treatment attempts. There are four major types of graft, which are commonly used for ACL reconstruction: Patellar, hamstring and quadriceps tendon autografts and allografts.

However, due to partially restricted clinical results as well as the legal situation in some countries the use of allografts is limited. The focus stays on autografts with a graft choice between patellar-, hamstring- and quadriceps tendon depending on patients factors and surgeons preferences as well as surgeons experiences [6, 20].

Hamstring tendons are the most commonly used graft in ACL reconstruction, which is due to the good biomechanical characteristics, the clinical outcomes and a reliable harvesting of the graft.

Quadriceps tendon grafts show excellent biomechanical qualities as well. Furthermore, the donor site morbidity is low and its use preserves the hamstring tendons as an ACL agonist [3, 29].

There are some cohort studies that compared knee stability and functional outcome scores of quadriceps- and hamstring tendon autograft, showing similar knee stability and functional outcome scores [7, 21, 29, 32].

To our knowledge only one randomized controlled trial comparing quadriceps- and hamstring tendon autograft has been published [38]. This study revealed similar clinical results and postoperative pain for both groups.

Therefore, high-level data comparing outcomes of quadriceps and hamstring tendon grafts for ACL reconstruction is sparse [35].

The goal of our study was to conduct a randomized, controlled two centre trial involving adults with an acute or chronic tear of the ACL to determine whether reconstruction with a hamstring tendon graft is superior to surgery using quadriceps tendon graft. We hypothesized that quadriceps tendon grafts would provide equal knee stability and comparable clinical results for the Lysholm and IKDC Score with respect to hamstring tendon grafts after 24 months of follow up.

## Methods

### Demographic data

**Fig. 1:**
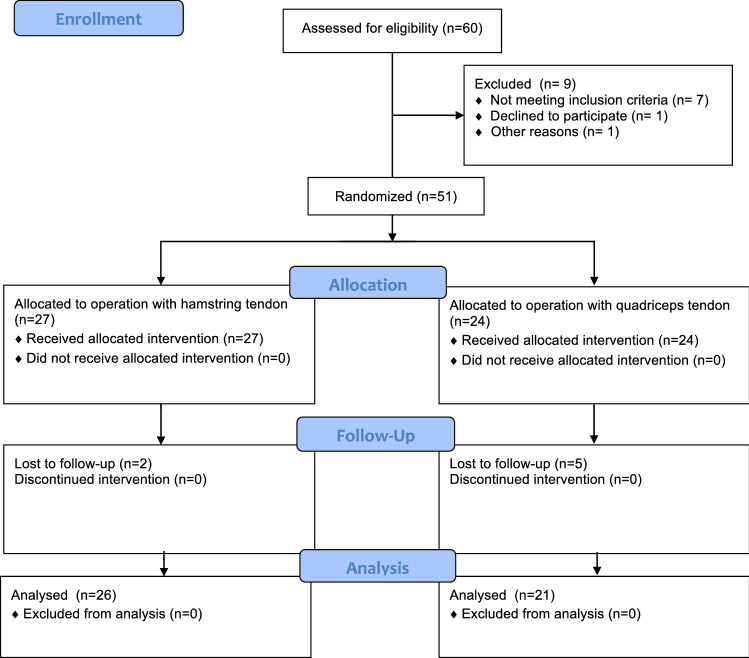
Consort flow chart: patient recruitment, allocation and follow up. The asymmetric allocation was caused by group randomization in blocks of 6

We conducted a two centre randomized controlled trial involving patients 18 years of age or older with a symptomatic anterior cruciate ligament tear. One blinded surgeon who was not participating during the surgical procedure investigated the patients. The knee incisions were covered by adhesive dressings before follow up. Randomization and reporting were done according to Consort requirements [23]. All eligible subjects were randomly assigned by computer generated random numbers in permuted blocks of 6 to undergo ACL reconstruction with either a hamstring tendon or quadriceps tendon graft. An investigator who was not involved in the randomization procedure prepared all sequentially numbered, opaque, sealed envelopes containing the assigned interventions to ensure that the sequence was concealed. We randomly assigned 27 patients to hamstring tendon reconstruction (H) and 24 to quadriceps tendon reconstruction (Q). Seven patients were lost to follow up. Graft rupture and other complications were recorded but kept for final analysis. See Fig. 1 Consort flow chart. Patients were recruited from December 2010 until June 2013. Details of the demographic data are shown in Table 1.

**Table 1:** Demographic data

Characteristic	Group H	Group Q	*P* value
*Baseline characteristics of the patients*			
No. of patients (centre A / centre B)	27 (12 / 15)	24 (10 / 14)	
Age (year)	32.7 ± 11.4	24.1 ± 3.6	0.001
Sex (Male/Female)	12/15	21/3	0.003
Weight (kg)	73.6 ± 11.3	79.0 ± 13.6	0.135
Height (cm)	171.2 ± 8.2	177.9 ± 7.3	0.003
Body-mass index	25.2 ± 4.0	24.9 ± 3.8	0.8
Smoking (yes/no)	6/21	6/18	1.0
Adequate trauma mechanism (%)	100.0%	88.9%	0.43
Time of surgery (minutes)	102.1 ± 37.6	112.5 ± 29.8	0.28
Graft size (mm)	7.9 ± 0.6	8.9 ± 0.5	0.001
*Concomitant injury*			
Med. collateral ligament laxity (0/1+/2+/3+))	(22/4/0/0)	(22/1/0/0)	0.35
Lateral collateral ligament laxity (0/1+/2+/3+)	(27/0/0/0)	(21/0/0/0)	1
Medial meniscus (with indication for meniscal repair)	5	3	0.28
Lateral meniscus (with indication for meniscal repair)	3	4	0.52
Duration of hospitalization (days)	3.8 ± 0.8	3.5 ± 1.1	0.47
Days from injury to surgery	203.9 ± 288.7	199.9 ± 300.0	0.97
Days with physiotherapy	152.1 ± 109.2	96.0 ± 67.3	0.22
Athlete (yes/no)	22/5	17/7	0.37

The local ethics committee approved the study, and informed consent was obtained from all participants. Inclusion as well as exclusion criteria are shown in Table 2.

**Table 2:** Display of inclusion and exclusion criteria

*Inclusion criteria:* Eligible subjects had ACL insufficiency as determined by subjective instability with giving way episodes, clinical examination, and MRI Scan Subjects were 18 to 50 years of age
*Exclusion criteria:* ACL insufficiency after reconstruction (revision surgery) Posterior cruciate ligament insufficiency Complete posterolateral corner injury Loss of more than two thirds of at least one meniscus during surgery Full-thickness cartilage lesion Fractures

### Surgical technique

Two senior knee surgeons, both with more than 10 years of experience in ACL reconstruction, performed all surgeries in two independent surgical centres.

Both surgeons used implant-free press-fit fixation techniques in a standardized fashion as described previously [10, 12, 13, 26]. Briefly, harvest of hamstring and quadriceps tendon was performed in a standard technique. Hamstring tendons were prepared as 4-strand grafts. A cortical-cancellous bone cylinder was sutured into the graft at the tibial end of the graft as a counter bearing [11]. The quadriceps tendon was harvested with a patellar bone block. The femoral tunnel was reamed in the size of the graft using the anteromedial portal technique. The tibial tunnel was similarly reamed using the size of the graft using a standard tibial aiming device. The femoral as well as the tibial tunnel was reamed with a hollow reamer. The cortico-cancellous bone plug, which was harvested before, was applied into the femoral tunnel to fix the graft in a press-fit technique [11]. A standard fluoroscopic lateral X-ray according to Bernard et al. [4] controlled all tunnel positions with a beath pin in centre A.

In both groups, a rehabilitation program with 3 weeks of partial weight bearing, immediate full range of motion and no hinge orthosis was used for all participants [7, 29]. COX-2 inhibitors were administered until pain levels were below a visual analogue scale (VAS) of 4 and then discontinued. Low molecular weight heparin was administered as the standard thromboprophylaxis over the time of partial weight bearing.

### Outcome measures

Patients were evaluated preoperatively and at 3, 6, 12 and 24 months after surgery. An independent examiner assessed all patients. At each visit, subjects completed the International Knee Documentation Committee (IKDC) evaluation form [28] and the Lysholm Score [5]. Side-to-side knee laxity was measured on manual maximum testing. Two arthrometers were used (KT-1000 arthrometer (MEDmetric Corp, San Diego, California) in centre A; Articometer (ARTICO Sportklinik, Germany) [19] in centre B). While the construction principle of the two arthrometers is similar, the only difference is that the Articometer measured digitally and the KT1000 measured in a mechanical setup. Strength measurement for extension and flexion in the knee was tested at each visit. Subjects were placed with hip flexed at 80°.

To determine the maximum strength using isokinetic testing, the most common method measuring extension and flexion strength of the lower limb was used [17, 18]. The setup included measurement of the point of rotation and lever arm. Knee strength was assessed using isokinetic parameters at angular velocities of 60°/sec (5 repetitions) with 3 sets and a one-minute break in between the sets. The peak torque value was determined. The testing was performed on a CON-TREX Multi-Joint System (CMV AG, Dübendorf Switzerland) [18]. The results were adjusted with the lever arm of the force and the body weight of the patients. The outcome is presented in Newton per kilogram body weight and compared to the strength of the healthy limb.

### Statistical analysis

For the determination of sample size, n-query Advisor 7.0 for Windows (Statcon, Witzenhausen, Germany) was used. The following parameters were chosen for the assessment of sample size: 2-side test, significance level of 0.05, difference in maximum manual knee laxity, difference in Lysholm score and difference in IKDC score of greater than 20% between the groups. For all of these parameters a sample size of 25 per group was sufficient to obtain a power of > 80% [16]. No interim analysis was performed. All reported P values are two-sided.

Baseline characteristics were analyzed by descriptive statistics. All mean values are reported with standard deviations. Analysis of binary and categorical variables between two groups was tested with the two-tailed Fishers exact test and the χ2-test. The 2 groups were compared using a 2-tailed Student’s *t* test for normal distribution. The Mann–Whitney U test was used as a nonparametric test. A two-sided *P* value of 0.05 was considered to indicate statistical significance. All reported P values are two-sided and were not adjusted for multiple comparisons. Statistical comparisons were made with the use of SPSS software (SPSS, Chicago, Illinois), version 24.

## Results

There was significantly improved knee stability, IKDC Score, Lysholm Score and pivot shift measurement at two-year follow-up. No significant difference was observed between the hamstring and quadriceps tendon group at any time. No significant difference between the two study groups was detected for return to work or sport. Complete data are shown in Table 3.

**Table 3:** Clinical results of the two study groups; data are reported as mean ± SD

	Hamstring group	Quadriceps group	*P* value
*Arthrometric side-to-side difference (KT 1000; injured—healthy)*			
Preoperative	4.8 ± 2.1	6.0 ± 2.9	0.116
2 year follow-up	0.2 ± 2.2	0.7 ± 1.1	0.643
*P* value	< .001	< .001	
*IKDC-score*			
Preoperative	59.0 ± 17.2	66.8 ± 16.9	0.109
2 year follow-up	83.7 ± 12.7	89.3 ± 12.2	0.169
*P* value	< .001	< .001	
*Lysholm-score*			
Preoperative	60.4 ± 18.5	72.3 ± 13.2	0.010
2 year follow-up	83.5 ± 17.4	90.4 ± 11.9	0.131
*P* value	< .001	< .001	
*Pivot Shift (Grade 0/1 + /2 + /3 +)*			
Preoperative	(3/10/5/1)	(3/5/9/0)	0.294
2 year follow-up	(26/0/0/0)	(23/0/0/0)	1
*P* value	< .001	< .001	
Return to work (in days)	75.2 ± 41.9	45.8 ± 42.7	0.16
Return to sport (in days)	95.2 ± 45.5	82.1 ± 45.6	0.62

### Strength for knee flexion and extension

**Fig. 2:**
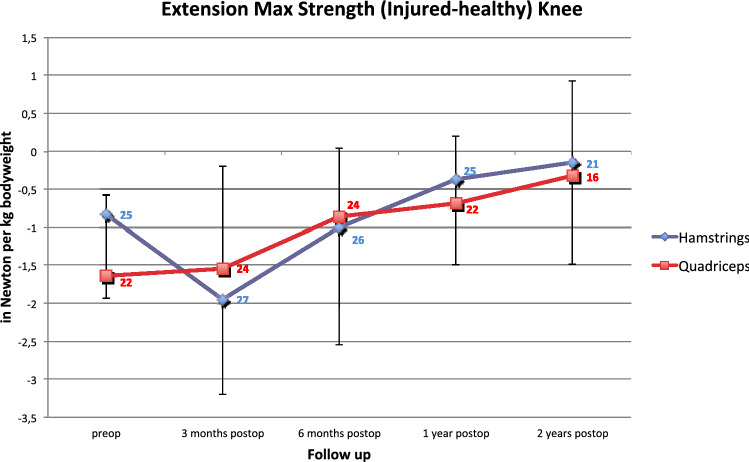
Extension Maximum Strength (injured–healthy knee extension) in Newton per kg bodyweight (mean ± SD, significance is marked with *) measured with isokinetic testing of the hamstring- and quadriceps tendon group (No. at data point represents the number of patients from preoperatively to two years of follow up)

The data of knee extension strength are shown in Fig. 2. No significant difference between group H and Q was noted.

**Fig. 3:**
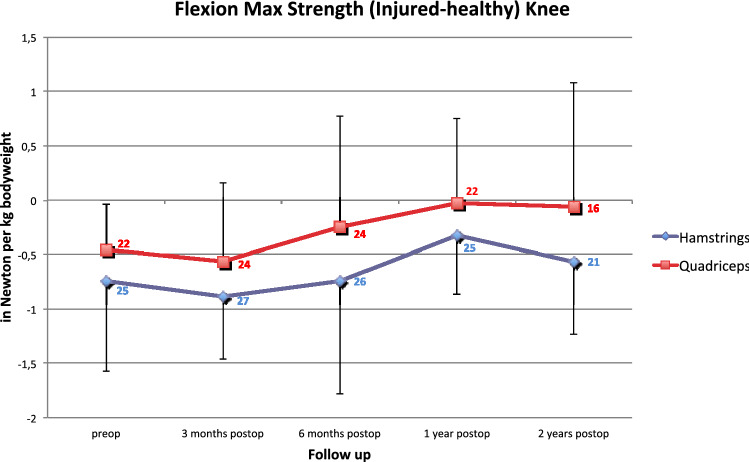
Flexion Maximum Strength (injured – healthy knee flexion) in Newton per kg bodyweight (mean ± SD, significance is marked with *) measured with isokinetic testing of the hamstring- and quadriceps tendon group (No. at data point represents the number of patients from preoperatively to two years of follow up)

The data of knee flexion strength are shown in Fig. 3. Similar to knee extension strength, no significant difference between the groups was observed.

### Adverse events

Clinical records were reviewed for all study visits. Anaesthesia and surgery records were retrieved for all surgical procedures, including the initial study treatment. There were six complications overall. In the hamstring tendon group, there was one graft retear, which was treated with revision ACL reconstruction with quadriceps tendon and one residual anteromedial knee instability, which was treated with reconstruction of the medial collateral knee ligament and revision of the ACL graft. In the quadriceps tendon group, there was one early infection, which was treated transplant retaining with antibiotics and arthroscopic lavage and three ACL graft retears, which were treated with ACL graft revision (two revision reconstructions with hamstring tendon graft and one healing response). The data of the two study groups did not differ significantly for retear rates or overall complications.

## Discussion

The most important finding of this study was that quadriceps tendon grafts yield comparably good results to hamstring tendon grafts in primary autograft ACL reconstruction at 2-year follow-up. No significant difference was found for IKDC- or Lysholm Score, anterior–posterior instability or Pivot shift.

There is no significant difference between quadriceps and hamstrings tendon graft fixation in a cadaveric study [9]. Ultimate failure loads for quadriceps and hamstring tendon grafts have been reported between 2352 and 4090 N.[25, 40]. Clinical data comparing quadriceps and hamstring tendon grafts are sparse. To the best of our knowledge, there is no randomized controlled trial comparing quadriceps tendon graft with hamstring tendon graft in primary ACL reconstruction with a follow up of two years. Nevertheless, there is a number of cohort studies which, compare quadriceps tendon and hamstring tendon reconstruction [7, 21, 29]. Besides the lower level of evidence, several differences of study design of these trials have to be taken into account. Contrary to our study, these former studies lack in strength testing and the presentation of side-to-side stability measured with arthrometer [29] or reporting about retears in their cohort [21]. A recently published randomized controlled trial, which compares quadriceps tendon autograft with hamstring tendon autograft reports similar clinical results and post-operative pain levels compared to our study [38]. However, this study has a sample size of only 28 analysed patients and only a 12-month follow-up [38]. In addition, no information on complications, strength and stability is provided.

Our study showed no significant difference between the two groups either, which is consistent with former studies. Different level I or II studies did not detect any difference in clinical performance no matter what treatment protocol was used [14, 28, 38, 41, 41, 42]. Our study showed that both surgical techniques result in a firm ACL reconstruction without a significant elongation of the graft during the observed time interval of 24 months. This was tested in centre A with the KT-1000 arthrometer, which is a commonly used instrument to measure the anterior–posterior laxity of the knee [1] and the Articometer [19] in centre B. Likewise the postoperative reduction of instability, measured by the antero–posterior translation, has previously been shown [21]. Biomechanical studies assume that there is a significant lengthening of the graft in the first couple of thousands cycles with medium load [31]. Similar to our trial, previous clinical studies detected no difference regarding side-to-side stability between the two graft types [7, 29]. Postoperative strength of extension of the injured knee is starting with a deficit, which is almost compensated towards the end of the 2-year follow-up time. In accordance with a previous study no extension strength deficit was found for quadriceps tendon grafts [7]. The greater postoperative weakness of knee extension in the injured limb compared to the healthy limb has been described previously [37]. Surprisingly, preoperative data in our study showed higher extension strength in the hamstring tendon group than in the quadriceps tendon group. Although previous data showed that relevant preoperative strength deficits lead to persisting reduction in strength for up to two years of follow up [8], our data did not confirm this finding. The postoperative strength rehabilitation developed similarly between the quadriceps and hamstring tendon group. An explanation for that result might either be measurement errors or, which is more likely, a randomization bias. The development of the flexion strength of the injured lower limb compared to the healthy limb did not differ significantly between the hamstring tendon group and the quadriceps tendon group. The postoperative strength showed a peak at the 1-year mark and dropped slightly, but not significantly, afterwards. These results are similar to previous trials [7, 21]. Consistently to the results by Lee et al., our data showed a slight but not significant weakness of the hamstring tendon group compared to the quadriceps tendon group in flexion strength recovery [21].

In cases of additional medial knee joint instability, it is desirable to preserve the hamstring tendons contributing to medial joint stability. Furthermore, some hamstring tendons might have previously been used for other reconstruction surgeries or might be too small in diameter to obtain a sufficient ACL graft. In these cases, the quadriceps tendon offers a promising alternative, providing sufficient graft thickness and avoiding weakening of medial joint stability [29].

There was a non-significant tendency towards more graft ruptures in centre B (three full thickness retears and one partial retear), compared with one graft elongation in a patient with recurrent anteromedial instability in centre A, leading up to an overall graft retear rate of 9.8%. The graft ruptures were divided into two hamstrings tendon ruptures and three quadriceps tendon ruptures. A possible reason might be the faster rehabilitation and earlier return to sport in centre B. The graft ruptures had all been adequate traumata with insufficient muscular performance, which are typical for young athletes returning back to competition too early. The overall rate of graft ruptures in our study seems to be slightly higher than in most previous studies [15, 27, 30]. However, a similar graft rupture rate of 9.4% has been reported previously [2].

Several limitations apply to this study. First, the relatively low number of patients failed to accomplish the determined sample size. Nevertheless, a high follow-up rate of 86% could be obtained. Second, randomization lead to three mismatches. The male/female ratio, the height of the patients and age differed significantly between the two groups. Owing to randomization, we had no control of the male/female ratio. It has been shown that the male/female ratio [38] as well as age of the patients [2] might have an impact on the outcome in ACL reconstruction. The graft size differed between the two groups, which might have been a result of the male/female mismatch as well. Retrospectively, the inclusion criteria for age could have been more strict. Furthermore, 5-strand or 6 strand hamstring autografts could have been used when undersized diameter grafts were harvested [22]. Compared to male patients, female patients reported significantly less extensor muscle strength and less improvement 1 year after ACL reconstruction [18], which could have had an impact on our strength results. Similarly, the significant difference of mean age between the two groups could as well have biased results [36, 39]. The height should not have an impact on the overall outcome, particularly as the BMI did not differ significantly [34].

Future randomized studies with an even-handed male/female ratio as well as balanced age groups should be conducted. Sufficient sample sizes and homogeneous surgical techniques are desirable to determine the optimal graft choice in primary ACL reconstruction.

## Conclusion

This prospective randomized controlled trial comparing quadriceps and hamstring tendon autografts in bone plug technique ACL reconstruction showed comparably good results in the Lysholm- and IKDC Score, anterior knee stability, strength for knee extension and flexion as well as return to work and sport at a follow-up of 24 months.

## Abbreviations

ACL: Anterior cruciate ligament
BMI: Body mass index
Centre A: Trauma Department, Hannover Medical School (MHH), Carl-Neuberg-Str. 1, 30625 Hannover, Germany
Centre B: ARTICO Sportklinik, Hirschbergstrasse 25, 78045 Villingen-Schwenningen, Germany
Group H: Group of patients operated with hamstring tendon graft
Group Q: Group of patients operated with quadriceps tendon graft
IKDC: International Knee Documentation Committee Score
Kg: Kilogram
MRI Scan: Magnetic resonance imaging
SD: Standard deviation
